# Panorama das Intervenções Coronárias Percutâneas em Oclusões Totais Crônicas em Centros Participantes do LATAM CTO Registry no Brasil

**DOI:** 10.36660/abc.20210462

**Published:** 2023-04-18

**Authors:** Antonio Carlos Botelho da Silva, João Eduardo Tinoco de Paula, Carlos M. Campos, Marcelo Harada Ribeiro, Evandro Martins, Marcos Danillo Peixoto Oliveira, Leandro Assumpção Côrtes, Aníbal Pereira Abelin, Cleverson Neves Zukowski, Gustavo Cervino Martinelli, Fábio Sândoli de Brito, Antônio José Muniz, Marcelo José de Carvalho Cantarelli, Pedro Beraldo de Andrade, César Rocha Medeiros, Breno de Alencar Araripe Falcão, Felipe Costa Fuchs, Leonardo Sinnott Silva, Tammuz Fattah, Ramiro Caldas Degrazia, José Armando Mangione, Cristiano Guedes Bezerra, Sandra Baradel, João Brum Silveira, Luiz Fernando Ybarra, Daniel Weillenmann, Carlos Gottschall, Viviana Lemke, Franciele Rosa da Silva, Marcia Moura Schmidt, Karlyse Claudino Belli, Pedro Piccaro de Oliveira, Alexandre Schaan de Quadros

**Affiliations:** 1 Hospital São José do Avaí Itaperuna RJ Brasil Hospital São José do Avaí, Itaperuna, RJ – Brasil; 2 Instituto Cardiovascular de Linhares UNICOR Linhares ES Brasil Instituto Cardiovascular de Linhares UNICOR, Linhares, ES – Brasil; 3 Hospital das Clínicas Faculdade de Medicina Universidade de São Paulo São Paulo SP Brasil Instituto do Coração do Hospital das Clínicas da Faculdade de Medicina da Universidade de São Paulo, São Paulo, SP – Brasil; 4 SOS Cardio Florianópolis SC Brasil SOS Cardio, Florianópolis, SC – Brasil; 5 Santa Casa de Misericórdia Maceió AL Brasil Santa Casa de Misericórdia, Maceió, AL – Brasil; 6 Hospital São Paulo Escola Paulista de Medicina UNIFESP São Paulo SP Brasil Hospital São Paulo – Escola Paulista de Medicina – UNIFESP, São Paulo, SP – Brasil; 7 Instituto Nacional de Cardiologia Rio de Janeiro RJ Brasil Instituto Nacional de Cardiologia, Rio de Janeiro, RJ – Brasil; 8 Instituto do Coração de Santa Maria Santa Maria RS Brasil Instituto do Coração de Santa Maria (ICOR), Santa Maria, RS – Brasil; 9 Rede D’Or Rio de Janeiro RJ Brasil Rede D’Or – Copa D’Or, Rio de Janeiro, RJ – Brasil; 10 Hospital Santa Izabel Santa Casa Misericórdia de Salvador Salvador BA Brasil Hospital Santa Izabel Santa Casa Misericórdia de Salvador, Salvador, BA – Brasil; 11 Hospital São Camilo São Paulo SP Brasil Hospital São Camilo, São Paulo, SP – Brasil; 12 Santa Casa de Misericórdia Juiz de Fora Juiz de Fora MG Brasil Santa Casa de Misericórdia Juiz de Fora, Juiz de Fora, MG – Brasil; 13 Hospital Leforte São Paulo SP Brasil Hospital Leforte, São Paulo, SP – Brasil; 14 Santa Casa de Marília Marília SP Brasil Santa Casa de Marília, Marília, SP – Brasil; 15 Hospital Badim Rio de Janeiro RJ Brasil Hospital Badim, Rio de Janeiro, RJ – Brasil; 16 Hospital de Messejana Fortaleza CE Brasil Hospital de Messejana, Fortaleza, CE – Brasil; 17 Hospital Mãe de Deus Porto Alegre RS Brasil Hospital Mãe de Deus, Porto Alegre, RS – Brasil; 18 Hospital Socimed Tubarão SC Brasil Hospital Socimed, Tubarão, SC – Brasil; 19 Instituto de Cardiologia do Estado de Santa Catarina São Jose SC Brasil Instituto de Cardiologia do Estado de Santa Catarina, São Jose, SC – Brasil; 20 Hospital Círculo Operário Caxiense Caxias do Sul RS Brasil Hospital Círculo Operário Caxiense, Caxias do Sul, RS – Brasil; 21 Hospital Nossa Senhora do Pompeia Salvador BA Brasil Hospital Nossa Senhora do Pompeia, Salvador, BA – Brasil; 22 Hospital Unimed Rio de Janeiro RJ Brasil Hospital Unimed, Rio de Janeiro, RJ – Brasil; 23 Beneficência Portuguesa de São Paulo São Paulo SP Brasil Beneficência Portuguesa de São Paulo, São Paulo, SP – Brasil; 24 Rede D’Or Hospitais Aliança, São Rafael e CardioPulmonar Salvador BA Brasil Rede D’Or – Hospitais Aliança, São Rafael e CardioPulmonar, Salvador, BA – Brasil; 25 Sociedade Brasileira de Hemodinâmica e Cardiologia Intervencionista São Paulo SP Brasil Sociedade Brasileira de Hemodinâmica e Cardiologia Intervencionista, São Paulo, SP – Brasil; 26 Centro Hospitalar e Universitário do Porto Hospital Santo Antôni Porto Portugal Centro Hospitalar e Universitário do Porto, Hospital Santo Antônio, Porto – Portugal; 27 London Health Sciences Centre Schulich School of Medicine and Dentistry Western University Ontario Canadá London Health Sciences Centre, Schulich School of Medicine and Dentistry, Western University, Ontario – Canadá; 28 Kantosspital St Gallen Cantão Suíça Kantosspital St Gallen, Cantão – Suíça; 29 Hospital das Nações Curitiba PR Brasil Hospital das Nações, Curitiba, PR – Brasil; 30 Instituto de Cardiologia do Rio Grande do Sul Porto Alegre RS Brasil Instituto de Cardiologia do Rio Grande do Sul, Porto Alegre, RS – Brasil; 31 Hospital Divina Providência Porto Alegre RS Brasil Hospital Divina Providência, Porto Alegre, RS – Brasil

**Keywords:** Doença da Artéria Coronariana, Intervenção Coronária Percutânea/tendências, Oclusão Coronária, Hospitais/tendências, Equipamentos e Provisões Hospitalares/tendências

## Abstract

**Fundamento:**

Tem sido observado um grande avanço nas técnicas e nos dispositivos para a realização de intervenções coronárias percutâneas (ICP) em oclusões totais coronarianas crônicas (OTC), mas existem poucos dados da prática do mundo real em países em desenvolvimento.

**Objetivos:**

Relatar as características clínicas e angiográficas, os aspectos dos procedimentos e os resultados clínicos da ICP de OTC em centros dedicados a esse procedimento no Brasil.

**Métodos:**

Os pacientes incluídos foram submetidos à ICP de OTC em centros participantes do LATAM CTO Registry, um registro multicêntrico latino-americano dedicado à coleta prospectiva desses dados. Os critérios de inclusão foram procedimentos realizados no Brasil, idade acima de 18 anos e presença de OTC com tentativa de ICP. A definição de OTC foi lesão de 100% em uma artéria coronária epicárdica, conhecida ou estimada como tendo pelo menos 3 meses de evolução.

**Resultados:**

Foram incluídos dados de 1.196 ICPs de OTC. Os procedimentos foram realizados principalmente para controle da angina (85%) e/ou tratamento de uma grande área isquêmica (24%). A taxa de sucesso técnico foi de 84% e foi alcançada com técnicas de fios anterógrados em 81%, dissecção/reentrada anterógrada em 9% e retrógrada em 10% dos procedimentos. Os eventos cardiovasculares adversos intra-hospitalares ocorreram em 2,3% dos casos, sendo a mortalidade de 0,75%.

**Conclusões:**

As OTC podem ser tratadas no Brasil por intervenção coronária percutânea de forma efetiva e com baixas taxas de complicações. O desenvolvimento científico e tecnológico observado nessa área na última década reflete-se na prática clínica de centros brasileiros dedicados a essa técnica.

## Introdução

A oclusão coronariana crônica (OTC) tem grande prevalência, sendo encontrada em aproximadamente um em cada três pacientes submetidos à angiografia coronária diagnóstica e podendo chegar até 50% em estudos de pacientes com cirurgia de revascularização do miocárdio (CRM) prévia. A OTC é uma das causas mais frequente de revascularização incompleta. As intervenções coronárias percutâneas (ICP) em OTC têm sido tradicionalmente associadas com menores taxas de sucesso e mais complicações quando comparadas às ICP em estenoses com fluxo anterógrado. Esses aspectos estão relacionados às dificuldades técnicas em cruzar a oclusão com o fio-guia, mas também à maior complexidade angiográfica, ao maior perfil de risco e à presença de comorbidades.^1-3^ Nos últimos anos, tem sido observado um grande avanço nas técnicas e nos dispositivos para realizar a recanalização de uma OTC, com taxas de sucesso próximas de 90% em registros internacionais de ICP em OTC.^4^ No entanto, existem poucos dados contemporâneos sobre as características e resultados desses procedimentos na realidade brasileira. O objetivo deste estudo foi descrever as características clínicas e angiográficas, os aspectos dos procedimentos, as complicações e os desfechos clínicos de pacientes com OTC contemporaneamente submetidos à ICP no Brasil em centros dedicados à ICP em OTC.

## Métodos

### Pacientes

Os pacientes incluídos neste estudo foram submetidos à ICP para o tratamento de OTC em hospitais brasileiros participantes do LATAM CTO Registry, sendo os critérios de inclusão idade acima de 18 anos e presença de OTC com tentativa de ICP, indicada por médico assistente. Não houve exigências de volume mínimo de procedimentos nos centros participantes. A definição de OTC foi lesão de 100% em uma artéria coronária epicárdica, conhecida ou estimada como tendo pelo menos 3 meses de duração.^5,6^ Todas as decisões referentes às indicações e ao tratamento clínico dos pacientes foram realizadas pelos médicos assistentes, sem interferência dos pesquisadores, com termo de consentimento livre e esclarecido, e o estudo foi aprovado pelo Comitê de Ética Institucional.

### Coleta e monitoramento dos dados

Os dados foram inseridos em um registro latino-americano multicêntrico de ICP em OTC iniciado pelo grupo de investigadores, com o apoio da Sociedade Brasileira de Hemodinâmica e Cardiologia Intervencionista. O banco de dados foi gerenciado pelo centro coordenador por meio da plataforma *Research Electronic Data Capture* (REDCap),^7^ ferramenta aprovada pela Agência Nacional de Vigilância Sanitária (Anvisa).

Todos os investigadores receberam um manual com instruções padronizadas para a inclusão dos dados nas planilhas eletrônicas. O material teve como foco os objetivos de cadastro, o esclarecimento do processo de coleta e o armazenamento dos dados (computadores, *tablets* e/ou celulares, de acordo com a demanda de cada centro participante). Os centros receberam suporte on-line e telefônico para dúvidas de inclusão ou conclusão de casos e para *feedbacks* mensais para dados faltantes e valores discrepantes. Foram utilizadas regras internas de qualidade para aprimorar a qualidade do banco de dados por meio de análises descritivas dos dados, que eram resumidas e enviadas no formato de relatórios mensais de monitoria para os centros participantes.

### Definições

A isquemia de grau moderado a severa foi definida como a presença de defeito perfusional por cintilografia, ecocardiografia de estresse ou ressonância magnética igual ou superior a 10%. A calcificação moderada/severa foi definida como um envolvimento de pelo menos 50% do vaso pela angiografia. A tortuosidade moderada/severa foi considerada quando eram observadas duas angulações de pelo menos 70º ou uma angulação de pelo menos 90º no vaso-alvo, no segmento proximal à OTC. Os cotos proximal e distal foram definidos como brusco ou afilado. Os vasos colaterais foram classificados como úteis para abordagem se considerados pelo operador como passíveis de cruzamento por um fio-guia e um microcateter e também através da classificação de Werner.^8^ As definições dos escores preditores de sucesso e complexidade J-CTO, PROGRESS, CL e ORA eram realizadas automaticamente conforme a inclusão das informações angiográficas e clínicas necessárias para seu cálculo.^9-12^

As seguintes estratégias foram consideradas para a realização dos procedimentos: (a) fios anterógrados: consistiu na tentativa de cruzar diretamente o segmento ocluído com o uso de diferentes fios-guia, de forma progressiva ou não; (b) dissecção e reentrada anterógrada: foi definida como um procedimento por abordagem anterógrada durante o qual o operador intencionalmente usou o espaço subintimal para superar parcial ou totalmente o segmento ocluído, com fios-guia ou dispositivos dedicados, reentrando no lúmen verdadeiro distalmente à oclusão; e (c) procedimento retrógrado: foi definido como uma tentativa de recanalização por um vaso colateral ou enxerto (ocluído ou não) que irriga um segmento distal à oclusão, podendo ser com técnicas de fios intraplacas ou dissecção e reentrada. O sucesso técnico foi definido como recanalização da OTC com implante de stent, fluxo final do Thrombolysis in Myocardial Infarction (TIMI) II/III e estenose residual menor que 30%. O sucesso do procedimento foi definido como a obtenção do sucesso técnico sem eventos cardiovasculares adversos maiores (ECAM).

### Desfechos clínicos e complicações

Definiu-se ECAM como a combinação de morte, infarto agudo do miocárdio (IAM) e acidente vascular cerebral (AVC). Caracterizou-se IAM de acordo com a versão mais recente da definição universal de IAM.^13^ O AVC foi definido como um novo déficit neurológico focal de início súbito e de causa irreversível presumivelmente cerebrovascular dentro de 24 horas e não causado por qualquer outra causa facilmente identificável.

As complicações do procedimento incluíram sangramento maior, perfuração coronária, tamponamento cardíaco e revascularização de urgência com ICP ou CRM. Um sangramento maior foi definido como qualquer sangramento com redução na hemoglobina > 3 g/dL, transfusão sanguínea ou intervenção cirúrgica. A perfuração coronária foi definida através da classificação de Ellis.^14^ O tamponamento cardíaco foi definido como comprometimento hemodinâmico causado por acúmulo agudo de sangue no espaço pericárdico. A revascularização de urgência foi definida como um procedimento realizado durante a internação sem planejamento prévio, para tratamento de angina e/ou isquemia recorrente.

### Análise estatística

Foi efetuada uma análise descritiva dos dados. As variáveis contínuas paramétricas foram apresentadas como média ± desvio-padrão (DP), as variáveis não paramétricas foram apresentadas como mediana (intervalo interquartil), e as variáveis categóricas, como frequências absolutas e relativas. Todas as análises foram realizadas no *software* SPSS, versão 27.0. O teste estatístico empregado para verificação da normalidade dos dados foi o de Kolmogorov-Smirnov. O nível de significância estatística adotado foi < 0,05.

## Resultados

Foram incluídos dados de procedimentos de 1.196 ICP em OTC realizadas em 26 hospitais brasileiros participantes do LATAM CTO Registry. A média de idade foi de 63,46±10,56 anos, sendo que a maioria dos pacientes era do sexo masculino, da raça branca e com diagnóstico de hipertensão arterial sistêmica (Tabela 1). Mais de 1/3 dos pacientes apresentava diagnóstico de diabetes melito, metade tinha histórico de IAM, e mais da metade apresentava revascularização miocárdica percutânea ou cirúrgica prévia. A fração de ejeção média do ventrículo esquerdo foi de 55,50±12,18%. O controle da angina foi a mais frequente indicação dos procedimentos (85%), seguida do tratamento de isquemia moderada/severa (24%).

**Tabela 1:** – Características clínicas dos pacientes (n = 1.196)

Idade, anos	63,46±10,56
Sexo masculino	887 (74%)
Raça branca	869 (77%)
Hipertensão arterial sistêmica	1.064 (90%)
Dislipidemia	816 (69%)
Tabagismo ativo	177 (15%)
Diabetes melito	462 (39%)
História familiar positiva	394 (35%)
**História médica pregressa**	
Infarto do miocárdio	529 (48%)
ICP	520 (47%)
Tentativa prévia de ICP em OTC	156 (13%)
Cirurgia de revascularização miocárdica	157 (14%)
Insuficiência cardíaca congestiva	132 (12%)
AVC	39 (3,5%)
Doença arterial periférica	142 (13%)
Doença renal crônica	82 (7,4%)
Fração de ejeção, %	55,50±12,18
**Indicações do procedimento**	
Controle da angina	1.010 (85%)
Isquemia moderada/severa	284 (24%)
Insuficiência cardíaca	120 (10%)
Arritmia ventricular	11 (0,9%)
Outros	22 (1,9%)

*ICP: intervenção coronária percutânea; OTC: oclusão total crônica; AVC: acidente vascular cerebral.*

Em relação aos medicamentos utilizados no momento da ICP de OCT, a grande maioria dos pacientes estava em uso de mais de um antianginoso, predominando betabloqueador. Além disso, a maioria também utilizava dupla antiagregação plaquetária, inibidor da enzima conversora da angiotensina e estatinas (Tabela 2).

**Tabela 2:** – Tratamento clínico antes da ICP de OTC (n = 1.196)

Estatina	1.034 (87,9%)
IECA	478 (40,6%)
ARA II	457 (38,9%)
AAS	1.094 (93,0%)
Outro antiplaquetário	825 (70,2%)
Betabloqueador	838 (71,3%)
Nitrato	415 (35,3%)
Bloqueador canais de cálcio	205 (17,4%)
Trimetazidina	50 (4,3%)
Cumarínico	3 (0,3%)
NOAC	7 (0,6%)

*AAS: ácido acetilsalicílico; IECA: inibidor da enzima conversora de aldosterona; ARA II: antagonista do receptor da angiotensina II; NOAC: novos anticoagulantes orais.*

A artéria descendente anterior e a artéria coronária direita foram os principais vasos-alvo, com comprimento médio das lesões de 25±15 mm e coto proximal em ponta de lápis na maioria das lesões (Tabela 3). Foi observada calcificação moderada/severa em aproximadamente 1/3 dos pacientes e ausência de colaterais e/ou enxertos adequados para abordagem retrógrada em 43% dos casos. O escore médio dos pacientes estudados, medido com o escore J-CTO, foi de 1,84±1,18.

**Tabela 3:** – Características angiográficas (n = 1.196)

Vaso-alvo	
Artéria descendente anterior	453 (39%)
Artéria coronária direita	476 (40%)
Artéria circunflexa	234 (20%)
Tronco da coronária esquerda	7 (0,6%)
Comprimento da lesão, mm	25,21±14,87
Coto proximal afilado	697 (60%)
Calcificação moderada/severa	176 (15%)
Tortuosidade moderada/severa	176 (15%)
Bifurcação no coto distal	360 (34%)
Ausência de colaterais para intervenção	493 (43%)
**Tamanho das colaterais (escore de Werner)**	
0	238 (21%)
1	572 (51%)
2	283 (25%)
Reestenose intra-stent	158 (14%)
**Escores de complexidade angiográfica**	
J-CTO	1,84±1,18
PROGRESS CTO	0,97±0,86
CL	2,81±1,57
ORA	1,04±0,68

**Figura 1:**
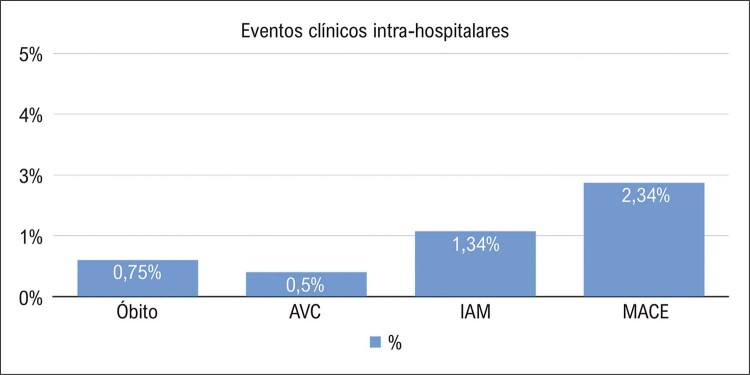
– Taxas de eventos clínicos em 30 dias na população do estudo. AVC: acidente vascular cerebral; IAM: infarto agudo do miocárdio; MACE: eventos cardíacos adversos maiores.

Na Figura Central, observamos as taxas de sucesso de registros internacionais gerais. A taxa de sucesso técnico nos centros brasileiros foi de 84%, e o sucesso do procedimento ocorreu em 82% dos casos. A estratégia com maior taxa de sucesso foi a de fios anterógrados, sendo que as abordagens de dissecção e reentrada anterógrada e retrógrada foram utilizadas em aproximadamente 10% dos casos cada. Um único acesso femoral ocorreu em 26% dos procedimentos, e um único acesso radial, em 20% dos procedimentos. A injeção contralateral foi utilizada em metade dos casos, sendo que a combinação de vias de acesso principal foi a radial e femoral (27%), seguida de dois acessos femorais (22%). Um microcateter foi utilizado em 3/4 dos procedimentos, sendo que o microcateter *Finecross*^*®*^ foi o mais frequente. Os fios-guia que mais frequentemente cruzaram as oclusões foram o *Whisper*^®^ e o *PT2*^®^, sendo que a mediana do tempo para cruzar a oclusão foi baixa, em torno de 15 minutos. Foram implantados em média 1,98±1,19 stents farmacológicos por procedimento (Tabela 4). As taxas de ECAM e complicações na amostra foram baixas, aproximadamente 2% (Figuras 1 e 2).

**Figura Central:**
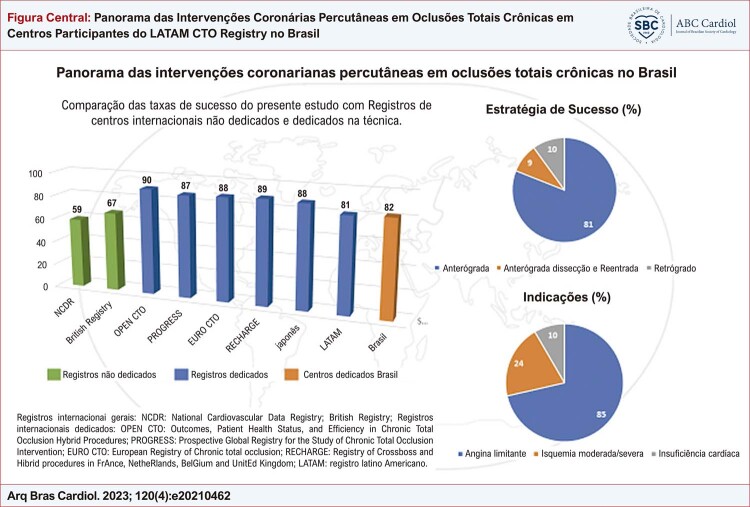
: Panorama das Intervenções Coronárias Percutâneas em Oclusões Totais Crônicas em Centros Participantes do LATAM CTO Registry no Brasil

**Tabela 4:** – Aspectos dos procedimentos (n = 1.196)

Via de acesso arterial	
Radial-femoral	322 (27%)
Femoral isolada	307 (26%)
Bifemoral	259 (22%)
Radial isolada	236 (20%)
Birradial	49 (4%)
Injeção contralateral	631 (54%)
Tempo de cruzamento	15 min (8-33 min)
**Fio-guia que cruzou a OTC**	
*Whisper*^®^	238 (28%)
*PT2*^®^	187 (22%)
*Fielder FC*^®^	63 (7,5%)
*Runthrough NS*^®^	45 (5,4%)
*Progress 80*^®^	41 (4,9%)
*ProVia 9*^®^	27 (3,2%)
*Progress 40*^®^	26 (3,1%)
*MiracleBros 3*^®^	23 (2,8%)
*Confianza Pro 12*^®^	21 (2,5%)
*Progress 200T*^®^	21 (2,5%)
Uso de microcateter	897 (75%)
**Tipos de microcateter**	
*Finecross*^*®*^	478 (44%)
Balão *over-the-wire*	147 (18%)
*Turnpike Spiral*^®^	85 (8,4%)
*Turnpike*^®^	73 (6,8%)
*Supercross*^®^	42 (4,1%)
Outro (n = 832)	173 (21%)
Número de balões/procedimento	3,04±3,48
Stents/procedimento	1,98±1,19
**Estratégia de sucesso**	
AW	795 (81%)
ADR	88 (9%)
Retrógrada	97 (10%)
Uso de estratégia retrógrada	166 (14%)
Aterectomia rotacional	28 (3%)
USIC	95 (10%)
Microcateter dedicado – *CrossBoss*^*®*^	36 (3,8%)
Balão dedicado – *StinGray*^*®*^	24 (2,6%)
Tempo de fluoroscopia, min	37,33±22,44
Volume contraste, mL	221,11±106,29
Sucesso técnico	84%
Sucesso clínico	82%

*ADR: dissecção e reentrada anterógrada; AW: fios-guia anterógrados; USIC: ultrassom intracoronário; OTC: oclusão coronariana crônica.*

**Figura 2:**
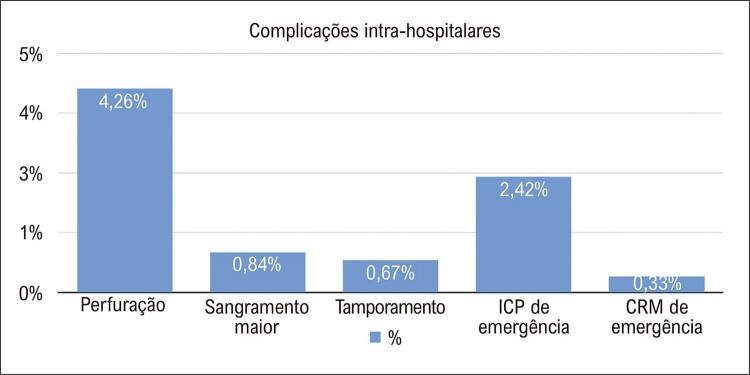
– Taxas de complicações intra-hospitalares na população do estudo. ICP: intervenção coronariana percutânea; CRM: cirurgia de revascularização do miocárdio.

## Discussão

Neste estudo, relatamos dados contemporâneas de ICP em OTC realizadas por centros brasileiros participantes do Registro Latino-Americano de OTC, incluindo dados clínicos e angiográficos, os aspectos do procedimento e os ECAM. Há amplo relato de dados desses procedimentos provenientes de países da América do Norte, Europa Ocidental e Japão,^1,15-18^ porém há uma escassez de informações em nosso meio. Nessa análise contemporânea representativa da prática brasileira, encontramos resultados encorajadores, com taxas de sucesso acima de 80% e baixas taxas de complicações e de ECAM. Também foi relevante a demonstração de que as principais indicações de realização das intervenções, angina e tratamento de isquemia miocárdica significativa, assim como o tratamento clínico recebido pelos pacientes antes das intervenções, estavam de acordo com as diretrizes.^15,16^ A presente análise é relevante por se tratar do primeiro relato da prática médica de centros de referência brasileiros no tratamento da ICP de OTC com as técnicas contemporâneas e a utilização das abordagens recomendadas pelos consensos internacionais.^17^

As indicações para realização de ICP de OTC foram recentemente questionadas por ensaios clínicos randomizados que não mostraram benefício desses procedimentos em redução de eventos cardiovasculares ou melhora da função ventricular, embora diversas limitações metodológicas tenham sido observadas nesses estudos.^19-21^ Por outro lado, a ICP em OTC melhorou significativamente os sintomas e a qualidade de vida quando comparada à terapia medicamentosa ideal em dois estudos randomizados recentes.^22,23^ Nesse contexto, é animador perceber que 85% dos procedimentos relatados no presente estudo tenham sido realizados para alívio de sintomas, refletindo uma grande aderência dos centros brasileiros às boas práticas clínicas e às diretrizes internacionais.

Embora a presença de uma OTC ocorra em até 18 a 52% das cineangiocoronariografias.^19,24,25^ a maioria dos casos não possui indicação clínica para intervenção. No entanto, um percentual desses pacientes apresenta angina significativa, refratária ao tratamento clínico e com comprometimento da qualidade de vida, ou necessita de intervenção na OTC como estratégia de revascularização completa em pacientes multiarteriais. Acreditamos que o presente relato demonstre a factibilidade de oferecer um tratamento efetivo para esses pacientes no Brasil com taxas satisfatórias de sucesso e baixo índice de complicações, em um contexto de mundo real.

Na Tabela 5, comparamos os resultados do tratamento de OTC em nosso meio com os registros contemporâneos da literatura, que categorizamos como Registros Nacionais de ICP em centros não selecionados quanto à *expertise* para ICP de OTC^26,27^ e Registros de ICP de OTC em centros dedicados a essas intervenções nos Estados Unidos, Europa, Japão e América Latina.^2,4,10,28-31^ Os dados dos registros de centros dedicados à ICP em OTC em países desenvolvidos demonstram taxas de sucesso superiores ao presente estudo, fato que pode ser explicado por terem sido realizadas por operadores de referência no contexto global e com ampla oferta de dispositivos e materiais para sua realização. Por outro lado, as taxas de sucesso em nosso meio, assim como no registro LATAM, foram superiores àquelas de Registros Nacionais de países desenvolvidos que não selecionaram somente centros dedicados à ICP de OTC, evidenciando já uma considerável *expertise* na realização de ICP em OTC em nosso meio.

**Tabela 5:** – Dados de Registros Nacionais de intervenções coronárias percutâneas (ICP) em centros não dedicados (ND) quanto à expertise para ICP de oclusão total crônica (OTC) e de Registros de ICP de OTC em centros dedicados (D) a essas intervenções nos Estados Unidos, Europa, Japão e América Latina e do presente estudo

	n	Sucesso	ECAM	Óbito
**Registros Nacionais ND**				
NCDR^26^	22.365	59%	1,6%	0,4%
British Registry (2014)Cardiovascular (27)	28.050	67%	0,73%	0,2%
**Registros de ICP de OTC D**				
OPEN CTO^28^	1.000	90%	7%	0.9%
PROGRESS^4^	3.055	87%	3%	0.85%
EURO CTO^2^	4.314	88%	0.5%	0.1%
RECHARGE^10^	1.253	89%	2.6%	0.2%
Japonês^30^	3.229	88%	0.5%	0.2%
LATAM^31^	1.040	81%	3%	1%
Brasil	1.196	82%	2,3%	0,75%

*ECAM: eventos cardiovasculares adversos maiores; NCDR: National Cardiovascular Data Registry; OPEN CTO: Outcomes, Patient Health Status, and Efficiency in Chronic Total Occlusion Hybrid Procedures; EURO CTO: European Registry of Chronic Total Occlusion; RECHARGE: REgistry of Crossboss and Hybrid procedures in FrAnce, NetheRlands, BelGium and UnitEd Kingdom; LATAM: Registro Latino-Americano.*

A complexidade das oclusões em nosso registro, conforme avaliada pela pontuação J-CTO, foi semelhante à de outros Registros dedicados a ICP de OTC, mas as informações dos Registros Nacionais de países desenvolvidos não estavam disponíveis. As taxas de complicações e ECAM em nosso meio também foram semelhantes entre os Registros dedicados ao tratamento de ICP de OTC. Essas comparações, embora coloquem em perspectiva a realidade brasileira e latino-americana com a prática médica de outros países, devem ser vistas com cautela em virtude de potenciais vieses na seleção de centros, aferição de eventos e outros fatores de confusão.

Conforme mencionado acima, nossas menores taxas de sucesso podem estar relacionadas a recursos mais limitados e diferentes fases da curva de aprendizado pelos centros e operadores participantes.^32^ Diferentemente de outros registros dedicados a ICP em OTC (Japão, Estados Unidos e Europa), em nosso meio e na experiência de centros da América Latina (da qual o Brasil também participa), não estabelecemos um número mínimo de casos por operador ou por centro para participar. Nosso objetivo foi mostrar um quadro da prática clínica em ICP de OTC no Brasil em serviços engajados no tratamento da OTC de forma dedicada, e todos os centros dispostos a participar foram incluídos. Nossos resultados podem, portanto, ser mais generalizáveis e representar a realidade da maioria dos serviços de cardiologia intervencionista com interesse em ICP de OTC.

O uso de microcateteres e injeções contralaterais são consideradas boas práticas em diversos documentos.^1,4,26,28^ O fato de apenas metade dos procedimentos em nosso estudo ter sido feita com injeção contralateral e apenas 2/3 usar microcateter pode refletir a curva de aprendizado em curso em nosso país. Embora um uso menor de microcateteres do que o recomendado possa estar relacionado a reembolso e questões financeiras, não existe esse problema em relação às injeções contralaterais, exceto a escolha do operador. Essas observações destacam a importância de uma educação continuada e treinamento adequado dos operadores dispostos a realizar ICP em OTC.

### Limitações

Os dados incluídos foram reportados pelos centros, sem auditoria externa ou monitoramento no local, mas verificamos periodicamente o banco de dados em busca de *outliers*, valores espúrios e assimetrias em um esforço para melhorar a qualidade dos dados. Além disso, um dicionário de dados e um manual de instruções detalhado foram enviados a todos os investigadores para padronizar a coleta e minimizar sua variabilidade, sendo que também oferecemos suporte continuado aos centros para esclarecimento de dúvidas e auxílio nas coletas. A inclusão de pacientes por cada centro não foi necessariamente consecutiva, não sendo possível excluir um potencial viés de seleção. As características angiográficas e do procedimento não foram avaliadas de forma independente por um laboratório central, o que também pode ser considerado uma limitação. A avaliação dos sistemas de pontuação (escores) depende muito da realização da angiografia dupla, e seu uso em apenas metade dos casos pode ter superestimado os escores. Os resultados clínicos não foram adjudicados centralmente por um comitê de eventos central, mas definições padronizadas foram fornecidas aos centros através do manual do estudo.

## Conclusões

As OTC podem ser tratadas no Brasil por ICP de forma efetiva, segura e com baixas taxas de complicações, em centros dedicados a esses procedimentos, refletindo o desenvolvimento científico e tecnológico observado nessa área na última década.
