# Supplementary material for: Panorama das Intervenções Coronárias Percutâneas em Oclusões Totais Crônicas em Centros Participantes do LATAM CTO Registry no Brasil
Source: Arq Bras Cardiol. 2023 Apr 18;120(4):e20210462. [Article in Portuguese] doi: 10.36660/abc.20210462 (PMC10263433; doi:10.36660/abc.20210462)
Supplement: Translation

# Translation: Overview of Percutaneous Coronary Interventions for Chronic Total Occlusions Treated at Brazilian Centers Participating in the LATAM CTO Registry

**Authors:** Antonio Carlos Botelho da Silva, João Eduardo Tinoco de Paula, Carlos M. Campos, Marcelo Harada Ribeiro, Evandro Martins, Marcos Danillo Peixoto Oliveira, Leandro Assumpção Côrtes, Aníbal Pereira Abelin, Cleverson Neves Zukowski, Gustavo Cervino Martinelli, Fábio Sândoli de Brito, Antônio José Muniz, Marcelo José de Carvalho Cantarelli, Pedro Beraldo de Andrade, César Rocha Medeiros, Breno de Alencar Araripe Falcão, Felipe Costa Fuchs, Leonardo Sinnott Silva, Tammuz Fattah, Ramiro Caldas Degrazia, José Armando Mangione, Cristiano Guedes Bezerra, Sandra Baradel, João Brum Silveira, Luiz Fernando Ybarra, Daniel Weillenmann, Carlos Gottschall, Viviana Lemke, Franciele Rosa da Silva, Marcia Moura Schmidt, Karlyse Claudino Belli, Pedro Piccaro de Oliveira, Alexandre Schaan de Quadros

**Affiliations:** 1 Brasil Hospital São José do Avaí, Itaperuna, RJ – Brasil; 2 Brasil Instituto Cardiovascular de Linhares UNICOR, Linhares, ES – Brasil; 3 Brasil Instituto do Coração do Hospital das Clínicas da Faculdade de Medicina da Universidade de São Paulo, São Paulo, SP – Brasil; 4 Brasil SOS Cardio, Florianópolis, SC – Brasil; 5 Brasil Santa Casa de Misericórdia, Maceió, AL – Brasil; 6 Brasil Hospital São Paulo – Escola Paulista de Medicina – UNIFESP, São Paulo, SP – Brasil; 7 Brasil Instituto Nacional de Cardiologia, Rio de Janeiro, RJ – Brasil; 8 Brasil Instituto do Coração de Santa Maria (ICOR), Santa Maria, RS – Brasil; 9 Brasil Rede D’Or – Copa D’Or, Rio de Janeiro, RJ – Brasil; 10 Brasil Hospital Santa Izabel Santa Casa Misericórdia de Salvador, Salvador, BA – Brasil; 11 Brasil Hospital São Camilo, São Paulo, SP – Brasil; 12 Brasil Santa Casa de Misericórdia Juiz de Fora, Juiz de Fora, MG – Brasil; 13 Brasil Hospital Leforte, São Paulo, SP – Brasil; 14 Brasil Santa Casa de Marília, Marília, SP – Brasil; 15 Brasil Hospital Badim, Rio de Janeiro, RJ – Brasil; 16 Brasil Hospital de Messejana, Fortaleza, CE – Brasil; 17 Brasil Hospital Mãe de Deus, Porto Alegre, RS – Brasil; 18 Brasil Hospital Socimed, Tubarão, SC – Brasil; 19 Brasil Instituto de Cardiologia do Estado de Santa Catarina, São Jose, SC – Brasil; 20 Brasil Hospital Círculo Operário Caxiense, Caxias do Sul, RS – Brasil; 21 Brasil Hospital Nossa Senhora do Pompeia, Salvador, BA – Brasil; 22 Brasil Hospital Unimed, Rio de Janeiro, RJ – Brasil; 23 Brasil Beneficência Portuguesa de São Paulo, São Paulo, SP – Brasil; 24 Brasil Rede D’Or – Hospitais Aliança, São Rafael e CardioPulmonar, Salvador, BA – Brasil; 25 Brasil Sociedade Brasileira de Hemodinâmica e Cardiologia Intervencionista, São Paulo, SP – Brasil; 26 Portugal Centro Hospitalar e Universitário do Porto, Hospital Santo Antônio, Porto – Portugal; 27 Canadá London Health Sciences Centre, Schulich School of Medicine and Dentistry, Western University, Ontario – Canadá; 28 Suíça Kantosspital St Gallen, Cantão – Suíça; 29 Brasil Hospital das Nações, Curitiba, PR – Brasil; 30 Brasil Instituto de Cardiologia do Rio Grande do Sul, Porto Alegre, RS – Brasil; 31 Brasil Hospital Divina Providência, Porto Alegre, RS – Brasil

**Keywords:** Coronary Artery Disease, Percutaneous Coronary Intervention/trends, Coronary Occlusion, Hospitals/trends, Equipment and Supplies Hospital/trends

## Abstract

**Background:**

Major advances have been seen in techniques and devices for performing percutaneous coronary interventions (PCIs) for chronic total occlusions (CTOs), but there are limited real-world practice data from developing countries.

**Objectives:**

To report clinical and angiographic characteristics, procedural aspects, and clinical outcomes of CTO PCI performed at dedicated centers in Brazil.

**Methods:**

Included patients underwent CTO PCI at centers participating in the LATAM CTO Registry, a Latin American multicenter registry dedicated to prospective collection of these data. Inclusion criteria were procedures performed in Brazil, age 18 years or over, and presence of CTO with PCI attempt. CTO was defined as a 100% lesion in an epicardial coronary artery, known or estimated to have lasted at least 3 months.

**Results:**

Data on 1196 CTO PCIs were included. Procedures were performed primarily for angina control (85%) and/or treatment of moderate/severe ischemia (24%). Technical success rate was 84%, being achieved with antegrade wire approaches in 81% of procedures, antegrade dissection and re-entry in 9%, and retrograde approaches in 10%. In-hospital adverse cardiovascular events occurred in 2.3% of cases, with a mortality rate of 0.75%.

**Conclusions:**

CTOs can be treated effectively in Brazil by using PCI, with low complication rates. The scientific and technological development observed in this area in the past decade is reflected in the clinical practice of dedicated Brazilian centers.

## Introduction

The prevalence of chronic total occlusion (CTO) is high, with approximately one in three patients undergoing diagnostic coronary angiography, and may reach up to 50% in studies of patients with previous coronary artery bypass grafting (CABG). The condition is also one of the most frequent causes of incomplete revascularization. Percutaneous coronary intervention (PCI) for CTO has been traditionally associated with lower success rates and more complications compared to PCI for stenosis and antegrade flow. This is related to technical difficulties in crossing occlusion with the guidewire but also to greater angiographic complexity, higher risk profile, and presence of comorbidities.^1-3^ In recent years, major advances in techniques and devices to recanalize CTOs have been seen, with international CTO PCI registries reporting success rates near 90%.^4^ However, there are limited contemporary data on the characteristics and outcomes of these procedures in Brazil. This study aimed to describe the clinical and angiographic characteristics, procedural aspects, complications, and clinical outcomes of contemporary patients with CTO undergoing PCI at Brazilian centers dedicated to CTO PCI.

## Methods

### Patients

Included patients underwent PCI for treatment of CTO at Brazilian hospitals participating in the LATAM CTO Registry. The inclusion criteria were being 18 years of age or over and having CTO with PCI attempt indicated by the attending physician. There were no minimum procedural volume requirements for participating centers. CTO was defined as a 100% lesion in an epicardial coronary artery, known or estimated to have lasted at least 3 months.^5,6^ All decisions regarding patient indications and clinical treatment were made by the attending physicians, with no interference from the researchers. Informed consent was obtained, and the study was approved by a research ethics committee.

### Data collection and monitoring

Data were included in a multicenter Latin American CTO PCI registry initiated by the group of investigators with support of the Brazilian Society of Hemodynamics and Interventional Cardiology. The database was managed by the coordinating center using Research Electronic Data Capture (REDCap),^7^ an application approved by the Brazilian Health Regulatory Agency (Anvisa).

All investigators received a manual with standardized instructions for entering data into electronic spreadsheets. The material focused on registry objectives, data collection, and storage processes (computers, tablets, and/or cell phones, according to the needs of each participating center). The centers received online and telephone support for questions regarding inclusion or completion of cases and monthly feedback for missing data and outliers. Internal quality rules were used to improve the quality of the database, consisting of descriptive analyses of the data, which were summarized and submitted as monthly monitoring reports to the participating centers.

### Definitions

Moderate-to-severe ischemia was defined as the presence of a perfusion defect detected by scintigraphy, stress echocardiography, or magnetic resonance imaging equal to or greater than 10%. Moderate/severe calcification was defined as at least 50% vessel involvement detected by angiography. Moderate/severe tortuosity was considered when two angulations of at least 70 degrees or one angulation of at least 90 degrees were observed in the target vessel, more specifically in the segment proximal to the CTO. Proximal and distal stumps were defined as blunt or tapered.Collateral vessels were classified as useful for the approach if considered, by the operator, crossable by a guidewire and a microcatheter, and also according to Werner classification.^8^ Scoring systems for predicting success and complexity — J-CTO, PROGRESS, CL, and ORA scores — were used automatically according to the inclusion of angiographic and clinical information required for their calculations.^9-12^

The following strategies were considered for performing the procedures: (a) antegrade wires consisted of an attempt to directly cross the occluded segment using different guidewires, either progressively or not; (b) antegrade dissection and re-entry (ADR) was defined as an antegrade procedure during which the operator intentionally used the subintimal space to partially or completely pass the occluded segment with guidewires or dedicated devices, re-entering the true lumen distally to the occlusion; and (c) retrograde procedure was defined as an attempt at recanalization using a collateral vessel or graft (either occluded or not) that irrigates a segment distal to the occlusion, which could be done using intraplaque wire techniques or dissection and re-entry. Technical success was defined as CTO recanalization with stent implantation, final TIMI grade II/III flow, and residual stenosis lower than 30%. Procedural success was the achievement of technical success without major adverse cardiovascular events (MACEs).

### Clinical outcomes and complications

MACE was defined as a composite of death, myocardial infarction (MI), and stroke. MI was characterized according to the most recent version of the universal definition.^13^ Stroke was defined as a new, sudden-onset focal neurological deficit of irreversible, presumably cerebrovascular cause within 24 hours and not caused by any other easily identifiable cause.

Procedural complications included major bleeding, coronary perforation, cardiac tamponade, and urgent revascularization with PCI or CABG. Major bleeding was defined as any bleeding with reduced hemoglobin > 3 g/dl, blood transfusion, or surgical intervention. Coronary perforation was defined according to the Ellis classification.^14^ Cardiac tamponade was defined as hemodynamic compromise caused by acute accumulation of blood in the pericardial space. Urgent revascularization was defined as an unplanned procedure performed during hospitalization for treatment of angina and/or recurrent ischemia.

### Statistical analysis

A descriptive analysis of the data was performed. Parametric continuous variables were reported as mean ± standard deviation (SD); nonparametric variables as median (interquartile range); and categorical variables as absolute and relative frequencies. All analyses were performed on SPSS 27.0. The Kolmogorov-Smirnov test was used to ascertain data normality. The significance level was set at <0.05.

## Results

Data on 1,196 CTO PCI procedures performed at 26 Brazilian hospitals participating in the LATAM CTO Registry were included. The mean age was 63.46±10.56 years, and most patients were male, White, and had a diagnosis of hypertension (Table 1). More than a third of patients had a diagnosis of diabetes mellitus, half had a history of MI, and more than half had previous percutaneous or surgical myocardial revascularization. The mean left ventricular ejection fraction was 55.50±12.18%. Angina control was the most frequent indication for procedures (85%), followed by treatment of moderate/severe ischemia (24%).

**Table 1:** – Patients’ clinical characteristics (n = 1196)

Age, years	63.46±10.56
Male	887 (74%)
White	869 (77%)
Hypertension	1,064 (90%)
Dyslipidemia	816 (69%)
Active smoking	177 (15%)
Diabetes mellitus	462 (39%)
Positive family history	394 (35%)
**Past medical history**	
Myocardial infarction	529 (48%)
PCI	520 (47%)
Previous attempt at CTO PCI	156 (13%)
Coronary artery bypass grafting	157 (14%)
Congestive heart failure	132 (12%)
Stroke	39 (3.5%)
Peripheral arterial disease	142 (13%)
Chronic kidney disease	82 (7.4%)
Ejection fraction, %	55.50±12.18
**Procedural indications**	
Angina control	1010 (85%)
Moderate/severe ischemia	284 (24%)
Heart failure	120 (10%)
Ventricular arrhythmia	11 (0.9%)
Other	22 (1.9%)

*PCI: percutaneous coronary intervention; CTO: chronic total occlusion.*

Regarding medications at the time of CTO PCI, the vast majority of patients were taking more than one antianginal drug, predominantly beta-blockers. Most were also on dual antiplatelet therapy, angiotensin-converting enzyme inhibitors, and statins (Table 2).

**Table 2:** – Clinical treatment before CTO PCI (n = 1196)

Statin	1034 (87.9%)
ACEI	478 (40.6%)
ARA II	457 (38.9%)
ASA	1094 (93.0%)
Other antiplatelet	825 (70.2%)
Beta-blocker	838 (71.3%)
Nitrate	415 (35.3%)
Calcium-channel blocker	205 (17.4%)
Trimetazidine	50 (4.3%)
Coumarin	3 (0.3%)
NOAC	7 (0.6%)

*AAS: acetylsalicylic acid; ACEI: angiotensin-converting enzyme inhibitor; ARA II: angiotensin II receptor antagonist; NOAC: novel oral anticoagulants.*

The left anterior descending artery and the right coronary artery were the most common target vessels, with a mean lesion size of 25±15 mm and a proximal stump shaped like a pencil tip in most lesions (Table 3). Moderate/severe calcification was observed in approximately one third of patients, and 43% had no collaterals and/or grafts suitable for a retrograde approach. The mean J-CTO score in the study patients was 1.84±1.18.

**Table 3:** – Angiographic characteristics (n = 1196)

Target vessel	
Left anterior descending artery	453 (39%)
Right coronary artery	476 (40%)
Circumflex artery	234 (20%)
Left main coronary artery	7 (0.6%)
Lesion size, mm	25.21±14.87
Tapered proximal stump	697 (60%)
Moderate/severe calcification	176 (15%)
Moderate/severe tortuosity	176 (15%)
Distal stump bifurcation	360 (34%)
No collaterals for intervention	493 (43%)
**Collateral channel size (Werner score)**	
0	238 (21%)
1	572 (51%)
2	283 (25%)
In-stent restenosis	158 (14%)
**Angiographic complexity scores**	
J-CTO score	1.84±1.18
PROGRESS CTO score	0.97±0.86
CL score	2.81±1.57
ORA score	1.04±0.68

The Central Figure shows success rates in general international registries. Technical success rate at Brazilian centers was 84%, and procedural success was 82%. The strategy showing the highest success rate was that of antegrade wires, with ADR and retrograde approaches being used in approximately 10% of cases each. A single femoral access was used in 26% of the procedures, and a single radial access in 20%. A contralateral injection was used in half of all cases, and the most commonly combined access sites were radial and femoral (27%), followed by bifemoral (22%). A microcatheter was used in three quarters of the procedures—the *Finecross*^®^ microcatheter was the most frequent. *Whisper*^*®*^ and *PT2*^*®*^ were the guidewires that most frequently crossed the occlusions, and the median crossing time was around 15 minutes, which is considered short. An average of 1.98±1.19 drug-eluting stents were implanted per procedure (Table 4). MACE and complication rates were approximately 2%, which is low (Figures 1A and 1B).

**Central Illustration:** : Overview of Percutaneous Coronary Interventions for Chronic Total Occlusions Treated at Brazilian Centers Participating in the LATAM CTO Registry

**Table 4:** – Procedural aspects (n = 1196)

Arterial access site	
Radial and femoral	322 (27%)
Femoral alone	307 (26%)
Bifemoral	259 (22%)
Radial alone	236 (20%)
Biradial	49 (4%)
Contralateral injection	631 (54%)
Crossing time	15 min (8-33 min)
**Guidewire crossing CTO**	
*Whisper*^*®*^	238 (28%)
*PT2*^*®*^	187 (22%)
*Fielder FC*^*®*^	63 (7.5%)
*Runthrough NS*^*®*^	45 (5.4%)
*Progress 80*^*®*^	41 (4.9%)
*ProVia 9*^*®*^	27 (3.2%)
*Progress 40*^*®*^	26 (3.1%)
*MiracleBros 3*^*®*^	23 (2.8%)
*Confianza Pro 12*^*®*^	21 (2.5%)
*Progress 200T*^*®*^	21 (2.5%)
Use of microcatheter	897 (75%)
**Types of microcatheter**	
*Finecross*^*®*^	478 (44%)
*Over-the-wire balloon*	147 (18%)
*Turnpike Spiral*^*®*^	85 (8.4%)
*Turnpike*^*®*^	73 (6.8%)
*Supercross*^*®*^	42 (4.1%)
Other (n = 832)	173 (21%)
Number of balloons/procedure	3.04±3.48
Stents/procedure	1.98±1.19
**Successful strategy**	
AW	795 (81%)
ADR	88 (9%)
Retrograde	97 (10%)
Use of retrograde strategy	166 (14%)
Rotational atherectomy	28 (3%)
IVUS	95 (10%)
Dedicated microcatheter – *CrossBoss*^®^	36 (3.8%)
Dedicated balloon – *StinGray*^®^	24 (2.6%)
Fluoroscopy time, min	37.33±22.44
Contrast volume, ml	221.11±106.29
Technical success	84%
Clinical success	82%

*ADR: antegrade dissection and re-entry; AW: antegrade wire; IVUS: intravascular ultrasound.*

**Figure 1:** – Thirty-day clinical event rates in the study population. MI: myocardial infarction; MACE: major adverse cardiovascular event.

## Discussion

In this study, we report contemporary CTO PCI data from Brazilian centers participating in the LATAM CTO Registry, including clinical and angiographic characteristics, procedural aspects, and MACEs. There is a broad collection of reports on these procedures from North America, Western Europe, and Japan,^1,15-18^ but Brazil still lacks information. In this contemporary analysis of Brazilian practice, we found encouraging results, with success rates above 80% and low rates of complications and MACEs. Also relevant was the demonstration that the main indications for performing interventions—angina control and treatment of significant myocardial ischemia—, as well as the clinical treatment received by patients before the interventions, were in accordance with current guidelines.^15,16^ The present analysis is the first medical practice report of Brazilian centers of excellence in CTO PCI using contemporary techniques and approaches recommended by international consensus groups.^17^

The indications for CTO PCI were recently questioned by randomized clinical trials that did not show the benefit of these procedures in reducing cardiovascular events or improving ventricular function, although several methodological limitations were observed in those studies.^19-21^ Conversely, CTO PCI significantly improved symptoms and quality of life compared to optimal drug therapy in two recent randomized trials.^22,23^ Therefore, we are encouraged to report that 85% of the procedures in the present study were performed to relieve symptoms, which reflects substantial adherence of Brazilian centers to good clinical practices and international guidelines.

Although CTO is found in up to 18-52% of coronary angiograms,^19,24,25^ most cases have no clinical indication for intervention. However, some of these patients have significant angina, refractory to clinical treatment and impairing quality of life, while those with multivessel disease require CTO intervention as a complete revascularization strategy. We believe that the present report demonstrates feasibility of providing an effective treatment for these patients in Brazil, with satisfactory success rates and low complication rates in a real-world context.

Table 5 compares CTO treatment outcomes in our setting with those of contemporary literature, which we categorized as national registries of nondedicated CTO PCI centers^26,27^ and registries of dedicated CTO PCI centers in the United States (US), Europe, Japan, and Latin America.^2,4,10,28-31^ Data from dedicated CTO PCI centers in developed countries demonstrate success rates higher than those of the present study, which can be explained by procedures being performed by experienced operators in the global context using a wide range of devices and materials. Conversely, success rates in our study, as well as in the LATAM registry, were higher than those of national registries of developed countries not restricted to dedicated CTO PCI centers, which shows considerable CTO PCI expertise in our setting.

**Table 5:** – Data from national registries of nondedicated (ND) CTO PCI centers and from registries of dedicated (D) CTO PCI centers in the USA, Europe, Japan, and Latin America, and from the present study

	n	Sucesso	ECAM	Óbito
**National registries**				
NCDR^26^	22.365	59%	1,6%	0,4%
British Registry (2014)Cardiovascular (27)	28.050	67%	0,73%	0,2%
**CTO PCI registries**				
OPEN CTO^28^	1.000	90%	7%	0.9%
PROGRESS^4^	3.055	87%	3%	0.85%
EURO CTO^2^	4.314	88%	0.5%	0.1%
RECHARGE^10^	1.253	89%	2.6%	0.2%
Japanese registry^30^	3.229	88%	0.5%	0.2%
LATAM^31^	1.040	81%	3%	1%
Brazil	1.196	82%	2,3%	0,75%

*CTO: chronic total occlusion; PCI: percutaneous coronary intervention; MACE: major adverse cardiovascular event; NCDR: National Cardiovascular Data Registry; OPEN CTO: Outcomes, Patient Health Status, and Efficiency in Chronic Total Occlusion Hybrid Procedures; EURO CTO: European Registry of Chronic total occlusion; RECHARGE: REgistry of CrossBoss and Hybrid procedures in FrAnce, NetheRlands, BelGium and UnitEd Kingdom; LATAM: Latin American registry.*

Occlusion complexity in our registry, as assessed by the J-CTO score, was similar to that of other dedicated CTO PCI registries, but information from national registries of developed countries was not available. The rates of complications and MACEs in our setting were also similar to those of dedicated CTO PCI registries. These comparisons, although putting Brazilian and Latin American practices into perspective against medical practice in other countries, should be viewed with caution because of potential biases in center selection, event measurement, and other confounding factors.

As we mentioned above, our lower success rates may be related to more limited resources and different stages of the learning curve shown by participating centers and operators.^32^ Unlike other dedicated CTO PCI registries (Japan, US, and Europe), we did not establish a minimum number of cases per operator or per center to participate in our study, similar to the experience of other Latin American centers. Our objective was to show an overview of CTO PCI practice at Brazilian services dedicated to CTO treatment, and all centers willing to participate were included. Our results may therefore be more generalizable and represent the reality of most interventional cardiology services that perform CTO PCI.

Microcatheters and contralateral injections are considered good practices in several reports.^1,4,26,28^ The fact that only half of the procedures in our study were performed with a contralateral injection and only two thirds used a microcatheter may reflect the ongoing learning curve in our country. Although the use of microcatheters below recommendations may be related to reimbursement and financial issues, this is not the case with contralateral injections, which are used at the discretion of the operator. These observations highlight the importance of continuing education and adequate training for operators willing to perform CTO PCI.

**Figure 2:** – In-hospital complication rates in the study population. PCI: percutaneous coronary intervention; CABG: coronary artery bypass grafting.

### Limitations

Included data were reported by the participating centers, with no external auditing or onsite monitoring, but we periodically checked the database for outliers, spurious data, and asymmetries in an effort to improve data quality. Additionally, a data dictionary and a detailed instruction manual were sent to all investigators to standardize data collection and minimize variability. We also provided continuous support to the centers that had questions and needed help with collections. Patient inclusion by each center was not necessarily consecutive; thus, we cannot exclude a potential selection bias. Angiographic and procedural characteristics were not independently evaluated by a central laboratory, which may also be considered a limitation. Evaluation of the scoring systems depends largely on the performance of dual injection angiography; however, this was used in only half of cases, which may have overestimated the scores. Clinical outcomes were not adjudicated centrally by a clinical event committee, but standardized definitions were provided to centers in the study manual.

## Conclusions

CTOs can be treated effectively and safely at Brazilian centers dedicated to CTO PCI, with low complication rates. This reflects the scientific and technological development observed in this area in the past decade.
